# Transactions of N. Y, State Med. Soc, New Hampshire Med. Society, and Penn. State Med. Society

**Published:** 1857-11

**Authors:** 


ART. VIII.—*Transactions of the Medical Society of the State of New New York. Feb.*, 1857.

*Transactions of the New Hampshire Medical Society. June*, 1857.

*Transactions of the Medical Society of the State of Pennsylvania. May*, 1857.

The transactions of state medical societies for the present year contain articles of more than ordinary interest, and are an indication of the steady advance which national medical science and literature are making in common with pursuits which more immediately engross the public attention. Notwithstanding the recent abridgement of the actual powers of medical societies, every high minded and honorable member of our profession must see that a gross violation of professional ethics brings its own reward; the respect and confidence of others, even of those who accept the sacrifice of professional standing, are not to be obtained by voluntarily relinquishing the good opinion of professional brethren, who have only the honor and dignity of their body at heart, and who are even more ready to defend and uphold each other in the right, than to censure the wrong. Our societies cannot be called powerless then, when we hold together its *honorable* members by such bonds.

The last meeting of the New York State Medical Society, commemorated the semi centennial epoch of its existence, and the address of Dr. March, president for 1856, contrasts the advance of the science, of agriculture, physics, etc., from a date of fifty years ago, with that of medical science. In the account of important discoveries in medicine and a comparison of them with discoveries in other departments, he shows that though the last half century has seen the most important and useful improvements and discoveries which have everywhere crowned the labors of men, we are by no means behind hand. In pathology, animal chemistry, minute anatomy and diagnosis, all of which have so important a bearing on therapeutics, we have made advances which are on a par with the discovery of steam power and the magnetic telegraph. The diminution of mortality is a sufficient indication of its actual benefit to the human race. Dr. March quotes the following statement: "Formerly, toward the middle of the last century, fifty or sixty out of one hundred children born in London, died before they had reached their fifth year of age; but the mortality has gradually and steadily diminished, so that now not above thirty or thirty-five in every hundred, die at that early period." Dr. March further states "that in the latter part of the sixteenth century, one-half of all who were born died under five years of age; the average longevity of the whole population was but eighteen years. In the seventeenth century one-half of the population died under twelve years of age. But in the first sixty years of the eighteenth century, one-half of the population lived over twenty-seven years. In the latter forty years one-half exceeded thirty-four years. At the beginning of the present century one-half exceeded forty years; and from 1838 to 1845, one-half exceeded forty-three years. The average longevity at the successive periods has been increased from eighteen years in the sixteenth century to 43 $\frac{7}{10}$ by the last reports."

The increase of advantages for prosecuting the study of medicine, as effected by the multiplication of medical schools and hospitals is also exhibited; and this interesting address closes by expressing the confident hope that at the end of another fifty years our successors may be able "to leave on record an account of far greater and more numerous achievements in the arts and sciences and advancements of our profession, than it has been our privilege to record."

The next is an interesting historical address by Dr. Willard, giving an account of the origin of the society fifty years ago, with sketches of the professional careers of the prominent physicians of New York State from Abraham Staats, surgeon, who emigrated from Holland in 1642, and settled at Fort Orange, to Dr. Joel A. Wing, whose likeness accompanies the present volume.

After an Eulogy on Samuel McClellan, M. D., prepared by Dr. Blatchford, a biographical sketch of the late Moses Hale, M. D., by Dr. Brinsmade; a biographical sketch of Dr. John McClellan, by Dr. Bates, and of Dr. Henry Mitchell, by Dr. Augustus Willard, which our limits compel us merely to mention, we have a paper on cholera-infantum, diarrhœa, and entero-colitis —their relation to each other—their pathology and treatment, by Edward H. Parker, M. D., of New York. In this paper the author attempts to disprove the position, that cholera infantum is a disease peculiar to this country. We know that cholera infantum is not a disease of the country districts, and that it selects for its victims (I say victims, for an examination of the vital statistics of large cities, shows an immense proportion of deaths from this malady,) the inhabitants of large cities, and children of parents who are compelled to live in unventilated and crowded situations, frequently giving their children insufficient nourishment, and that not of the best quality. These causes we know to exist in European cities to as great, if not a greater extent than here, and the author is disposed to think that the disease described by West as "inflammatory diarrhœa," and by others as "entero-colitis," is in some of its forms identical with the cholera infantum of this country. The author states that "neither writer uses the word cholera infantum, but West calls it inflammatory diarrhœa." It is true that in the first edition of his work on children, West makes no mention of the disease by the name of cholera infantum; but in his second edition, 1854, in speaking of the occasional rapid termination of the disease, he states: "This rapidly fatal termination is far from unusual in some of the Southern States of America, where diarrhœa, under the variaus names of *cholera infantum*, summer complaint, or gastro-follicula enteritis, annually destroys many thousands of children." Thus we have a direct acknowledgment of what Dr. Parker labors to prove. After an account of the mortality of this disease in different parts of the United States, the author discusses the point of a pathology distinct from diarrhœa and entero-colitis, he assumes that it is difficult or impossible to mark the line when an aggravated diarrhœa becomes a cholera infantum, or wherein lies the difference between it and an acute and sudden attack of entero-colitis. Diarrhœa is one of the chief symptoms of cholera infantnm, so it is often marked in typhoid fever, but because typhoid fever was accompanied by diarrhœ, we would not call the disease by a name which applied only to a prominent symptom.

Again, entero-colitis is, no doubt, frequently the result of cholera infantum, but it is shown by the highest authorities that it frequently occurs as a disease, and that probably in the first stages of cholera infamtun, it is not present, as there we frepuently have no febrile movement whatever. Several other interesting points relating to the pathology of this disease are discussed in this paper, but our space forbids us to notice them. The article is valuable as a contribution to an interesting subject which is hardly sufficiently spoken of in the books, and as recommending a course of treatment as perfect as any which has been recommended.

There are many other interesting reports in this volume which we are compelled to pass by.

The first article in the Transactions of the Penn. Medical Society, is the Annual Address, by its president, Dr. R. La Roche.

Anything eminating from the graceful pen of Dr. La Roche is sure to repay an attentive perusal, as much for the pleasure which we experience from contrasting its chaste and elegant style with the dry and formal details which we are compelled to undergo in many medical productions, as for the information and sound sense which we always find connected with it. But we were hardly prepared for the great pleasure which we experienced by a careful reading of this address, and regret that we are not able to give our readers the benefit of a fuller and more comprehensive review.

After commenting on the advantages which individuals derived from meeting together to discuss matters of common professional interest, especially those who were so situated as to be cut off from constant mingling with brother practitioners, thereby lacking "the vivifying influence of emulation," the author mentions the immediate object of the organization of the body which he was then addressing, which was the production of harmony among the rival medical factions of Philadelphia. He draws a graphic picture of the social relations which existed between the Philadelphia physicians forty years ago: street fights, the weekly meetings of the medical society, at which "the science of Billingsgate seemed to have taken precedence of all others," insulting attacks in the public journals, medical and secular, and other proceedings of a kindred character, which have been the source of so much ridicule from time immemorial. This society was then organized by the late Dr. Samuel Brown, and established by the initiation of four physicians, Prof. Jackson, Prof. Meigs, Dr. Thos. Harris, of the navy, and Dr. La

Roche. "A promise, modeled in a great measure on the oath of Hippocra tos, was exacted of each member at the moment of his admission — to obtain which an unanimous vote of those present was necessary. By this promise the member bound himself to live in peace and harmony with, and to do everything in his power to promote the welfare of his brethren in and out of the society, and to abide implicitely by a stringent code of ethics that had been prepared for our guidance."

This organization produced the desired effect, all difficulties were adjusted, and science moreover was advanced by the spirit of emulation which was excited among its members. The society then answered its purpose, and now stands as a monument that doctors can be made to agree, in a measure at least.

The doctor now proceeds to deprecate the opinion among most persons, and not a few members of our profession, concerning *practical* men so called, and men who are interested in the advancement of science sufficiently to wish to know what is done every day for its promotion; men who are called book-learned, theoretical, etc., but not practically equal to the emergencies which we meet at the bed side. Nothing could be more appropriate than the remarks of Dr. La Roche on this point: would that every physician and every patient could read and appreciate them. Not only in regard to every day practice and practitioners are these remarks applied, but to men of genius who might become lights and ornaments to the profession. He refers here to the expressed views of Sprengel on this subject: "When the observer, says the great historian of medicine, whatever be the extent of his genius, has nevertheless not enough of erudition to be acquainted with the observations of his predecessors, he runs the risk of repeating what has already been said a hundred times before, and of publishiug it as his own discovery." The Dr. then draws a faithful picture of the difficulties which a medical practitioner has to encounter, the annoyances to which he is subjected, and the length of time which must generally elapse before he can gain the confidence of the community, which is often when he has arrived at an age when he is hardly physically able to undergo the fatigues to which he is subjected. "How can he sustain himself under the influence of those depressing circumstances; how can he emerge, as it were, from obscurity, and contribute to the improvement of the science which he cultivates, unless he be buoyed up by a more than usual degree of energy of character and of professional zeal?" It is this zeal, this enthusiasm which he must have. The "Diable du corps — the sacred fire — the demon of genius." It is this enthusiasm which has impelled our great men to devote their lives to industrious seeking after knowledge; it is this which has prompted the noble acts of self-sacrifice to which Dr. La Roche alludes, and of which we are justly so proud, the devotion of physicians during the plague in olden times. "The two surgeons left at Jaffa by Bonaparte, to attend the pestiferous soldiers, whom he was forced to abandon." The "noble army of martyrs" who sacrificed themselves at Norfolk; with numerous other instances which show the self devotion of all high-minded physicians to their profession and to their fellow creatures.

The present policy of medical schools is then canvassed, the want of more thorough examination of candidates; and especially the absence of requirement of at least a fair education on the part of students, before they are permitted to enter the medical profession. He thinks that many young men are now drawn from less intellectual employments which are better adapted to their education and mental endowments, to the profession of medicine, by the cheap and especially free schools which are scattered over the country. No one can deny that the ranks of the profession are filled with men who would, perhaps, have made excellent cobblers or tailors, but who in their present position only contribute to reduce the standard of the profession and bring upon it popular discredit; these men have taken advantage of some neighboring country and, perhaps, free school, to employ the winter, when they have nothing else to do, to become *doctors* forsooth, and such doctors.

We cannot forbear quoting, before closing this notice, some of the remarks of Dr. La Roche on quackery as it exists in this country. In speaking of quackery in Europe as compared with the evil in this country, he says:

"But neither in England nor on the continent of Europe, are the members of the medical profession elbowed and circumvented and openly branded, as it were, by such low-bred, ignorant, and unprincipled quacks, as those by whom we are here infested. Nowhere is the market so overstocked with secret and other quack nostrums. The quack doctors in England are for the most part licensed graduates, who have taken to the nefarious practices after obtaining a diploma in some regular medical school. They are, of course, either fools or rogues, but still they are not uneducated men, and there is less disgrace in being placed on an equal footing with them by the public, than we are made to experience in this land of liberty. Here a physician—even of the highest eminence—is regarded in a professional and social point of view, not only as an erring graduate, but as the most arrant empiric and ignorant booby and rogue that may be pleased to assume the office and title of doctor."

Again, in speaking of the opinions of quacks as received by the public, he says: "Their opinions on professional subjects are cited in opposition to those of eminent practitioners and profound pathologists. Their testimony on points of medical jurisprudence, involving the reputation, liberty, and life of individuals, is received in courts of justice with as much deference as that of the most learned physicians and medical jurists."

How true is all this, and how few are they among our readers who have not experienced the annoyances and degradations to which we are subjected by being classed with such as are here described. The task of throwing off the incubus of quackery which weighs upon our country, seems hopeless indeed when some of the most intelligent persons (in other matters) still sanction it with their countenance and confidence. Were we rid of one form, we would have another, and we may now expect at any moment another monstrous humbug to supplant homœopathy, which now is on the decline.

*The Report of a Committee on Vaccination and Vaccine Virus.* This report, as well as a report on the same subject in the Transactions of the New York State Medical Society, recommends strongly the more general employment of vaccination and revaccination, and urges the necessity of some legislative action in the matter which will make it required by law as much as is possible; certainly some of the superfluous public funds could not be employed for a nobler purpose.

The rest of the transactions is composed of reports of country medical societies, containing articles of interest and importance, but which we cannot notice much in detail.

In the report of the Blair County Medical Society, we notice a report of the introduction of typhoid fever into a previously healthy district, where there can be no doubt but that it was propagated by contagion. The details very much resemble an account of the importation of typhoid fever into the town of Boston, twenty miles from Buffalo, mentioned by Prof. Flint in his work on Continued Fever, and was reported to the American Journal in 1845.

The Transactions of the New Hampshire Medical Society, contains the Annual Address, by the president, Francis P. Fitch, M. D., on the Want of Sanitary Knowledge; Report of Committee on Surgery, by Geo. H. Hubbard, M. D.; Nursing Sore Mouth, by Dr. Pray, orator for 1857; Report on Practical Medicine, by Wm. H. Brown, M. D.; The Contributions of Quackery to True Medical Science, by Thos. H. Marshall, M. D.; Report of the Corresponding Secretary, Levi G. Hill, M. D.; and Miscellaneous Observations in Obstetrics and Diseases of Women, by William Henry Thayer, M. D.

These reports are interesting and of value. The report on surgery, by Dr. Hubbard, shows to a gratifying extent the improvements in conservative surgery during the last few years. Our object is now to save limb as well as life, and injuries are left to nature which were formerly sure to be remedied by the knife.

Dr. Pray's paper on nursing sore mouth, is worthy of an attentive perusal.

The report of Dr. Brown on practical medicine, is very brief, and strikes us as being a little behind the age. The furious attacks which he deems it necessary to make upon a patient laboring under pneumonia or pleuritis, diseases which, in their uncomplicated form, it is now established are rarely fatal, is rather uncalled for, as his complaint is more his misfortune than his fault. F.

